# Global Mortality Estimates for the 2009 Influenza Pandemic from the GLaMOR Project: A Modeling Study

**DOI:** 10.1371/journal.pmed.1001558

**Published:** 2013-11-26

**Authors:** Lone Simonsen, Peter Spreeuwenberg, Roger Lustig, Robert J. Taylor, Douglas M. Fleming, Madelon Kroneman, Maria D. Van Kerkhove, Anthony W. Mounts, W. John Paget, the GLaMOR Collaborating Teams

**Affiliations:** 1 Department of Global Health, George Washington University School of Public Health and Health Services, Washington, District of Columbia, United States of America; 2 Sage Analytica, Bethesda, Maryland, United States of America; 3 Netherlands Institute for Health Services Research, Utrecht, Netherlands; 4 Royal College of General Practitioners, London, United Kingdom; 5 Medical Research Council Centre for Outbreak Analysis and Modelling, Department of Infectious Disease Epidemiology, Imperial College, London, United Kingdom; 6 Global Influenza Programme, World Health Organization, Geneva, Switzerland; University of Oxford, United Kingdom

## Abstract

**Background:**

Assessing the mortality impact of the 2009 influenza A H1N1 virus (H1N1pdm09) is essential for optimizing public health responses to future pandemics. The World Health Organization reported 18,631 laboratory-confirmed pandemic deaths, but the total pandemic mortality burden was substantially higher. We estimated the 2009 pandemic mortality burden through statistical modeling of mortality data from multiple countries.

**Methods and Findings:**

We obtained weekly virology and underlying cause-of-death mortality time series for 2005–2009 for 20 countries covering ∼35% of the world population. We applied a multivariate linear regression model to estimate pandemic respiratory mortality in each collaborating country. We then used these results plus ten country indicators in a multiple imputation model to project the mortality burden in all world countries. Between 123,000 and 203,000 pandemic respiratory deaths were estimated globally for the last 9 mo of 2009. The majority (62%–85%) were attributed to persons under 65 y of age. We observed a striking regional heterogeneity, with almost 20-fold higher mortality in some countries in the Americas than in Europe. The model attributed 148,000–249,000 respiratory deaths to influenza in an average pre-pandemic season, with only 19% in persons <65 y. Limitations include lack of representation of low-income countries among single-country estimates and an inability to study subsequent pandemic waves (2010–2012).

**Conclusions:**

We estimate that 2009 global pandemic respiratory mortality was ∼10-fold higher than the World Health Organization's laboratory-confirmed mortality count. Although the pandemic mortality estimate was similar in magnitude to that of seasonal influenza, a marked shift toward mortality among persons <65 y of age occurred, so that many more life-years were lost. The burden varied greatly among countries, corroborating early reports of far greater pandemic severity in the Americas than in Australia, New Zealand, and Europe. A collaborative network to collect and analyze mortality and hospitalization surveillance data is needed to rapidly establish the severity of future pandemics.

*Please see later in the article for the Editors' Summary*

## Abstract 2

Lone Simonsen and colleagues use a two-stage statistical modeling approach to estimate the global mortality burden of the 2009 influenza pandemic from mortality data obtained from multiple countries.

*Please see later in the article for the Editors' Summary*

## Editors' Summary

**Background:**

Every winter, millions of people catch influenza—a viral infection of the airways—and hundreds of thousands of people (mainly elderly individuals) die as a result. These seasonal epidemics occur because small but frequent changes in the influenza virus mean that the immune response produced by infection with one year's virus provides only partial protection against the next year's virus. Influenza viruses also occasionally emerge that are very different. Human populations have virtually no immunity to these new viruses, which can start global epidemics (pandemics) that kill millions of people. The most recent influenza pandemic, which was first recognized in Mexico in March 2009, was caused by the 2009 influenza A H1N1 pandemic (H1N1pdm09) virus. This virus spread rapidly, and on 11 June 2009, the World Health Organization (WHO) declared that an influenza pandemic was underway. H1N1pdm09 caused a mild disease in most people it infected, but by the time WHO announced that the pandemic was over (10 August 2010), there had been 18,632 laboratory-confirmed deaths from H1N1pdm09.

**Why Was This Study Done?:**

The modest number of laboratory-confirmed H1N1pdm09 deaths has caused commentators to wonder whether the public health response to H1N1pdm09 was excessive. However, as is the case with all influenza epidemics, the true mortality (death) burden from H1N1pdm09 is substantially higher than these figures indicate because only a minority of influenza-related deaths are definitively diagnosed by being confirmed in laboratory. Many influenza-related deaths result from secondary bacterial infections or from exacerbation of preexisting chronic conditions, and are not recorded as related to influenza infection. A more complete assessment of the impact of H1N1pdm09 on mortality is essential for the optimization of public health responses to future pandemics. In this modeling study (the Global Pandemic Mortality [GLaMOR] project), researchers use a two-stage statistical modeling approach to estimate the global mortality burden of the 2009 influenza pandemic from mortality data obtained from multiple countries.

**What Did the Researchers Do and Find?:**

The researchers obtained weekly virology data from the World Health Organization FluNet database and national influenza centers to identify influenza active periods, and obtained weekly national underlying cause-of-death time series for 2005–2009 from collaborators in more than 20 countries (35% of the world's population). They used a multivariate linear regression model to measure the numbers and rates of pandemic influenza respiratory deaths in each of these countries. Then, in the second stage of their analysis, they used a multiple imputation model that took into account country-specific geographical, economic, and health indicators to project the single-country estimates to all world countries. The researchers estimated that between 123,000 and 203,000 pandemic influenza respiratory deaths occurred globally from 1 April through 31 December 2009. Most of these deaths (62%–85%) occurred in people younger than 65 years old. There was a striking regional heterogeneity in deaths, with up to 20-fold higher mortality in Central and South American countries than in European countries. Finally, the model attributed 148,000–249,000 respiratory deaths to influenza in an average pre-pandemic season. Notably, only 19% of these deaths occurred in people younger than 65 years old.

**What Do These Findings Mean?:**

These findings suggest that respiratory mortality from the 2009 influenza pandemic was about 10-fold higher than laboratory-confirmed mortality. The true total mortality burden is likely to be even higher because deaths that occurred late in the winter of 2009–2010 and in later pandemic waves were missed in this analysis, and only pandemic influenza deaths that were recorded as respiratory deaths were included. The lack of single-country estimates from low-income countries may also limit the accuracy of these findings. Importantly, although the researchers' estimates of mortality from H1N1pdm09 and from seasonal influenza were of similar magnitude, the shift towards mortality among younger people means that more life-years were lost during the 2009 influenza pandemic than during an average pre-pandemic influenza season. Although the methods developed by the GLaMOR project can be used to make robust and comparable mortality estimates in future influenza pandemics, the lack of timeliness of such estimates needs to be remedied. One potential remedy, suggest the researchers, would be to establish a collaborative network that analyzes timely hospitalization and/or mortality data provided by sentinel countries. Such a network should be able to provide the rapid and reliable data about the severity of pandemic threats that is needed to guide public health policy decisions.

**Additional Information:**

Please access these websites via the online version of this summary at http://dx.doi.org/10.1371/journal.pmed.1001558.

- The US Centers for Disease Control and Prevention provides information about influenza for patients and professionals, including archived information on H1N1pdm09
- Flu.gov, a US government website, provides access to information on seasonal and pandemic influenza H1N1pdm09
- The World Health Organization provides information on influenza and on the global response to H1N1pdm09, including a publication on the evolution of H1N1pdm09 (some information in several languages). Information on FluNet, a global tool for influenza surveillance, is also available
- Public Health England provides information on pandemic influenza and archived information on H1N1pdm09
- More information for patients about H1N1pdm09 is available through Choices, an information resource provided by the UK National Health Service
- More information about the GLaMOR project is available

## Introduction

Recurring seasonal influenza epidemics impose a moderate, if variable, mortality burden every year. But when a new human-transmissible influenza virus emerges, the ensuing pandemic can be catastrophic; the 1918 Spanish influenza pandemic, for example, killed approximately 1%–2% of the global population. Understanding the global mortality impact of pandemic influenza—who died, where, and when—is fundamental to understanding how pandemics emerge and evolve, and will help to guide responses to future pandemics. And because so few pandemics have occurred in the modern era, it is essential that each one be studied thoroughly—even if, as was the case with the 2009 influenza A H1N1 pandemic (H1N1pdm09), the catastrophe failed to appear.

As of 31 August 2010 the World Health Organization (WHO) received reports of 18,449 laboratory-confirmed deaths from H1N1pdm09 infection [1]. This modest number has caused many to wonder what all the excitement was about, and some to question whether the pandemic response was excessive [2],[3]. But what is not widely appreciated is that the laboratory-confirmed total greatly underestimates the mortality burden, because only a minority of influenza-related deaths are ever definitively diagnosed as such. Additional influenza deaths result from secondary bacterial infections and exacerbation of preexisting chronic conditions, but are not recorded as being in any way related to influenza infection.

Statistical methods are therefore used to separate the influenza-attributable fraction of deaths from the background [4],[5]. These methods involve modeling seasonal cyclical patterns in mortality time series compiled from vital statistics, often coupled with viral surveillance data to provide information on the timing of influenza circulation. Vital statistics data usually contain information about cause of death, allowing researchers to estimate influenza-attributable “excess” deaths in broad categories such as pneumonia, respiratory, or cardiorespiratory deaths during influenza periods. The influenza-related excess in respiratory deaths can be measured with higher precision than the less specific all-cause and cardiorespiratory categories. However, because some deaths triggered by influenza are recorded as having been caused by underlying non-respiratory causes such as heart attack, stroke, diabetes, or chronic kidney conditions, analysis of the broader cardiorespiratory and all-cause categories typically captures more completely the influenza-related burden.

The majority of deaths from seasonal influenza occur among people aged 65 y or older, but in a pandemic the proportion of deaths among the young increases [4],[6]–[8]. Single-country studies of H1N1pdm09 mortality, using various cause-of-death outcomes and modeling techniques [9]–[14], have repeatedly documented such an age shift. For example, a comprehensive hospital-based sentinel surveillance study by Liang and colleagues found that in China only 4% of cases, 8% of hospitalizations, and 23% of pandemic deaths occurred in persons over 50 y of age [15]. On a global level, Van Kerkhove et al. reported a median age of 46 y among fatal laboratory-confirmed cases [16]. McCallum et al. reported that in the Western Pacific region during 2009, only 1% of laboratory-confirmed cases and 13% of laboratory-confirmed deaths were among persons 65 y of age or older [17].

The Global Pandemic Mortality (GLaMOR) project aimed to make a conservative estimate of the global H1N1pdm09 mortality burden in 2009 using statistical models applied to mortality, virology, and other available data. The project was funded by WHO, which requested global and regional estimates of H1N1pdm09 influenza deaths for the year 2009; thus, all mentions of pandemic flu mortality refer specifically to deaths that occurred in the last 9 mo of 2009. We invited global collaborators to contribute national mortality data detailed by week, age, and cause of death for 2005 through 2009, at minimum.

Our novel method was inspired by a study that estimated the 1918–1920 global pandemic mortality burden using a two-stage statistical approach [18]. In Stage 1, we used detailed time series of national mortality and virology data from collaborating teams to estimate the 2009 pandemic respiratory mortality in each collaborating country/administrative region. Each participating GLaMOR team included influenza experts with whom the core GLaMOR team discussed Stage 1 mortality estimates in detail. The Stage 1 model and results were made available to each collaborating team to encourage individual country publications of national burden estimates. In Stage 2, we used a hierarchical multiple imputation model that used geographical, economic, and health country indicators to project the Stage 1 single-country estimates to all world countries, and summed to obtain regional and world estimates (Figure S2) .

A previous study of global H1N1pdm09 mortality, published in 2012 by Dawood et al. [19], was conducted before 2009 mortality data became available. To overcome that problem, these authors implemented a probability model that took into account symptomatic attack rates and case fatality ratios measured in a set of wealthier countries, then used a “respiratory mortality multiplier” based on pre-2009 data to adjust for differences in respiratory disease fatality rates in different parts of the world. In contrast, we used national vital statistics data for 2009 to measure the actual pandemic mortality and its age patterns in countries representing ∼35% of the global population, then estimated the burden in the remaining countries using a novel projection method. As a result, while our global burden estimate is comparable to that of Dawood et al. [19], the regional pattern we found, with the Americas hit hard and Europe largely spared, corresponds more closely to what was reported as the pandemic unfolded. In addition, our method simultaneously generates pre-pandemic global seasonal influenza mortality estimates that we have presented here for comparison.

## Methods

### Data Sources and Preparation

We obtained weekly virology data from the WHO FluNet [20] to identify influenza active periods (some collaborators provided more detailed virology data). We created 3-wk moving averages of these data with a lag of 1 wk, to achieve the clinically observed lag of 1 wk from disease onset to death. We inspected the data for weeks in which the reported count dropped dramatically coincident with a major holiday; in three cases (Spain, Mexico, and Japan) we replaced the reported number with the average of positive counts in the two surrounding weeks. In most cases we encountered insufficient subtyping of influenza A viruses. Age-specific virology data were not available.

We requested weekly national mortality time series based on the “underlying” cause-of-death determination from 1 January 2005 to 31 December 2009, stratified by at least two age groups (<65 and ≥65 y) and by four International Classification of Diseases–10 (ICD-10)–coded outcomes: all causes, cardiorespiratory (J and I codes), respiratory (J codes), and pneumonia and influenza (codes J10–J18) (Table 1). Contributing countries/administrative zones represented ∼35% of the global population. To prepare the time series mortality data for modeling, we created 3-wk moving averages after removing data in affected countries from summer weeks with a documented heat wave or armed conflict with significant mortality. We de-trended the time series using a spline factor modeled from summer periods. The exclusions and summer periods are given in Table 2. We obtained age distribution data for H1N1pdm09 laboratory-confirmed deaths from collaborators in a subset of GLaMOR countries. Because of concerns about sharing data, collaborators in the UK and China ran the GLaMOR SAS code on their own data. As only aggregate (de-identified) national summary mortality data were used in this study, it was exempt from human subjects regulations.

**Table 1:** Participating countries for which GLaMOR estimated all-cause, cardiorespiratory, or respiratory pandemic-associated mortality (Stage 1) or which were used to evaluate performance of global projection methods (Stage 2).

WHO Region (Number of Countries)	Country	Income Level^a^	Data Years	Outcome^b^	Detail^c^	Virology Source	Percent World Population
**Countries for which GLaMOR made all-cause, cardiorespiratory, or respiratory Stage 1 estimates**							
**Africa (1)**	South Africa	Upper middle	2003–2009	AC,CR,R	Monthly	FluNet	0.7
**Eastern Mediterranean (0)**							
**Europe (9)**	Denmark	High	1998–2009	AC	Weekly	FluNet	0.1
	Israel	High	2004–2009	AC,CR,R^d^	Weekly	Israel	0.1
	France	High	1998–2009	AC,CR,R	Weekly	FluNet	0.9
	Germany	High	1998–2009	AC,CR,R	Weekly	Germany	1.3
	Poland	Upper middle	2003–2009	AC,CR,R	Weekly	FluNet	0.4
	Romania	Upper middle	2005–2009	AC,CR,R	Weekly	EuroFlu	0.3
	Slovenia	High	2003–2009	AC,CR,R	Weekly	FluNet	0.1
	Spain	High	2000–2009	AC,CR,R	Weekly	Spain	0.7
	UK	High	2000–2009	AC,CR,R	Weekly	UK	0.9
**Americas (4)**	Argentina	Upper middle	2001–2009	AC,CR,R	Monthly	FluNet	0.6
	Chile	Upper middle	2002–2009	AC,CR,R	Weekly	FluNet	0.2
	Mexico	Upper middle	2000–2009	AC,CR,R	Weekly	FluNet	1.6
	US	High	2000–2009	AC,CR,R	Weekly	FluNet	4.6
**South-East Asia (0)**							
**Western Pacific (7)**	Australia	High	2003–2009	AC,CR,R	Weekly	FluNet	0.3
	China^e^	Lower middle	2004–2009	AC,CR,R	Weekly	China	19.5
	Hong Kong	High	1999–2009	AC,CR,R	Weekly	Hong Kong	0.1
	Japan	High	1998–2009	AC,CR,R	Weekly	FluNet	1.9
	New Zealand	High	2000–2009	AC,CR,R	Weekly	FluNet	0.1
	Republic of Korea	High	2003–2009	CR,R	Weekly	FluNet	0.7
	Singapore	High	2007–2009	CR,R	Weekly	Singapore, FluNet	0.1
**Validation countries to evaluate performance of Stage 2 projection methods**							
**Americas (3)**	Brazil	Upper middle	NA	R	Weekly	NA	2.8
	Canada	High	NA	R	Weekly	Canada	0.5
	Peru	Upper middle	NA	R	Monthly	Peru	0.4
**Europe (1)**	Netherlands^f^	High	NA	R	Weekly	Netherlands	0.2
**South-East Asia (1)**	Bangladesh^g^	Low	NA	R	NA	Bangladesh	2.1

a Income level in 2009 [28].

b Underlying cause of mortality: AC, all cause; CR, cardiorespiratory (ICD-10 I and J codes); R, respiratory (ICD-10 J codes).

c Standard request was for age groupings: 0–4, 5–14, 15–44, 45–64, 65–84, and ≥85 y of age.

d Did not include influenza with pneumonia.

e Data from multiple surveillance settings representing rural and urban areas across China [14].

f Respiratory mortality estimated by Netherlands team based on estimated all-cause pandemic mortality [13].

g Respiratory mortality estimated by Bangladesh team using a novel method combining virology surveillance and verbal autopsy data [45].

NA, not available.

**Table 2:** Stage 1 customizations and all-ages model fit for 20 GLaMOR participating countries.

Country	Exclusions	Summer Definition (Week Numbers)	Secular Model Fit (*R* ^2^)	Full Model Fit (*R* ^2^)	Pandemic Parameter *p*-Value
Argentina	None	1–10,45–52	0.8290	0.9301	<0.0001
Australia	None	1–18,46–52	0.8657	0.9098	0.1206
Chile	None	1–17,42–52	0.7032	0.9128	<0.0001
China	None	—	—	—	<0.0001
France	Heat waves^s^	May–Sep	0.6989	0.8830	0.3836
Germany	Heat waves^s^	19–45	0.6684	0.9196	0.2918
Hong Kong	None	None	0.6098	0.7266	<0.0001
Israel	2006 Lebanese war	15–42	0.6832	0.9246	0.2718
Japan	None	27–40	0.7926	0.8813	0.0527
Mexico	None	Jun–Aug	0.7497	0.8500	<0.0001
New Zealand	None	1–19,43–52	0.7122	0.8320	0.0176
Poland	None	18–41	0.7096	0.8660	0.1634
Republic of Korea	None	23–43	0.6739	0.8320	0.0749
Romania	None	19–45	0.8047	0.8738	0.0054
Singapore	None	None	0.5306	0.6676	0.3705
Slovenia	None	17–44	0.5101	0.7270	0.4362
South Africa	None	1–14,39–52	0.8193	0.9254	<0.0001
Spain	Heat waves^a^	21–39	0.6640	0.8973	0.8243
UK	None	NA	0.7190	0.8756	0.1505
US	None	23–39	0.8086	0.9408	<0.0001

a Europe experienced severe heat waves in 2003 and 2006; hence, summer weeks with elevated mortality were excluded.

NA, not available.

When choosing which outcome to use as our primary estimate of mortality, we had to make a trade-off between sensitivity and specificity while maintaining sufficient precision. Modeling all-cause mortality data would by definition ensure that all deaths are captured (100% sensitivity), but would sacrifice specificity and therefore precision. At the other extreme, pneumonia and influenza (P&I) is a specific influenza outcome but captures only a fraction of total pandemic deaths.

After much deliberation and advice from the Ad Hoc Advisory Committee on H1N1pdm09 Mortality Estimates [21], we focused on respiratory deaths in order to provide a minimum estimate of pandemic mortality burden with reasonable confidence limits. This was necessary as we had found that it was difficult to tease out any influenza-attributable increase in all-cause mortality in most countries.

### Stage 1: Single-Country Pandemic Mortality Estimates

We developed a multivariate linear regression model of influenza burden based on correlations between laboratory surveillance and national mortality data [22]–[25]. The model included virology surveillance time series data, as well as terms for linear, square, cubic, and cyclical secular trends. We applied the same model form to each country. Where possible, the analysis was performed for four different mortality outcomes: all-cause deaths, respiratory and cardiovascular deaths, respiratory deaths, and deaths due to pneumonia and influenza. Data collection was carried out between 1 July 2011 and 31 March 2012. Stage 1 estimates were comparable in that we used standard ICD-10 definitions of mortality outcomes for each cause and applied the same multivariable linear regression model. We made estimates of respiratory and cardiorespiratory mortality for 20 countries, and estimates of all-cause mortality for 19 countries. Only estimates made with the GLaMOR model were used in the Stage 2 projections.

The GLaMOR Stage 1 model form was:
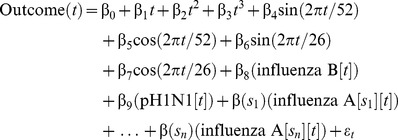
(1)where *t* is the running week variable, and the linear and polynomial terms track remaining secular trends. The cyclical terms (as whole- and half-year cycles) tracked seasonal mortality patterns from other respiratory pathogens and factors such as temperature and relative humidity. Each influenza A season had its own variable, which we set to 0 in all other seasons. This way, case fatality in individual seasons is allowed to vary. Models of this type usually use separate terms for influenza A subtypes because the mortality burden of H3N2 is known to be far greater than that of seasonal H1N1; however, because adequate subtyped laboratory surveillance data were not available from many Stage 1 countries, we instead introduced into the model separate influenza A terms for each season. Collaborators supplied data for a variable number of seasons (*s*_1_ to *s_n_*), with β(*s*_1_) to β(*s_n_*) the respective coefficients. Most collaborators supplied five seasons; thus, the model had ∼14 explanatory factors and ∼250 observed data points for most countries. The H1N1pdm09 season was defined as the weeks beginning 5 April through 27 December 2009, inclusive. For Hong Kong, Japan, and South Africa, where H3N2 co-circulated with the pandemic virus, we included separate time series of H1N1pdm09 and H3N2 samples during the pandemic period. We did not remove nonsignificant terms from the model. Because of a lack of respiratory syncytial virus virology data, we did not control for respiratory syncytial virus co-circulation.

We computed the pandemic attributions as the sums of the products of the pandemic model parameter β_9_ multiplied by the H1N1pdm09 positive count. When negative parameter values were obtained, mortality burden estimates were set to zero (as negative burden is not biologically meaningful). The confidence intervals were derived from uncertainty on the pandemic model parameter estimate β_9_. We determined the Stage 1 95% confidence intervals from the standard error on the H1N1pdm09 parameter estimate. We did not address autocorrelation in the residuals, and therefore the confidence intervals are narrow. We evaluated the model fit by three criteria: (1) the adjusted *R* ^2^ for the full model, (2) the “lift” achieved by adding the virology terms to a base model of only secular and cyclical terms, and (3) the significance of the pandemic term β_9_ in the model. We used SAS 9.2 for all Stage 1 analyses.

### Choosing among Four Stage 2 Global Projection Strategies

We explored four strategies to project our single-country estimates to the rest of the world before settling on multiple imputation, a Monte Carlo method that imputes values to missing data points and is often used to supply missing values in survey and census data [26].

#### 1. Survey method

The survey method was a direct extrapolation of the average Stage 1 pandemic mortality rates using bootstrapping, in which the average excess mortality rate and upper and lower 95% confidence levels were used to calculate the global numbers and rates of pandemic deaths by age group and WHO region.

The limitation of this method is that it assumes that the average pandemic mortality experience in the Stage 1 countries is representative of the experience in all countries, and gives only a global estimate (no country- or region-specific estimates).

#### 2. Gross national income/latitude method

The gross national income (GNI)/latitude method was derived from the method Murray et al. employed to estimate the 1918 global pandemic burden [18]. Influenza-attributable mortality was based on the relationship between measured pandemic mortality and per capita GNI and geographical latitude. Ordinary least squares regression models were used to relate the Stage 1 estimates to GNI and absolute latitude after natural logarithmic transformation of GNI and the dependent variable. The relationship was then used to estimate mortality in all world countries.

The limitation of this method is that the model assumes that GNI and latitude are sufficient proxies for the many variables that influence influenza mortality, and that mortality and the reasons for its variability in Stage 1 countries are representative for all countries. Importantly, there was an assumption that the relationship between GNI and excess mortality is exponential, so that the method yields very high mortality rates for low-income countries (e.g., countries in Africa).

#### 3. Matching method

The matching method was devised by the GLaMOR team. It obtains the missing data points by matching (as closely as possible) Stage 1 countries to non–Stage 1 countries based on a set of country indicators. It involves two steps: (1) a data creation step using the matching approach, and (2) a data analysis step where a hierarchical linear random effects model is used to provide a single estimate for each country.

The data creation step involves calculation of multiple estimates per country based on the indicators listed in Table 3. We chose these indicators because they could reasonably be expected to affect pandemic mortality and were available for all countries in the world from public domain sources [27]–[30]. WHO region and latitude reflect the differing epidemiology of seasonal influenza in the differing regions of the world. Age group all-cause mortality rates, population density, physician density, and rural population percent reflect both the access to health care and the likelihood of influenza transmission. Population age structure reflects the documented age-related impact of influenza virus infections. The prevalence of comorbidities (i.e., obesity, HIV, and tuberculosis) is likely to be correlated with a greater probability of a severe outcome of influenza infection. The mortality estimates in the Stage 1 countries were examined and matched, for each indicator, to countries in which the mortality was not known.

**Table 3:** Country indicators used as factors in the Stage 2 model that projects the measured Stage 1 pandemic and seasonal influenza mortality estimates to global and regional estimates.

Indicator Number	Indicator
1	WHO region (Africa, the Americas, Eastern Mediterranean, Europe, South-East Asia, Western Pacific)
2	Age group all-cause mortality rates (0–14, 15–59, 60+ y) [29]
3	Physician density (per 10,000 population) [27]
4	Obesity (percent with body mass index >30 kg/m^2^) [27]
5	Population density (per km^2^) [28]
6	Major infectious diseases (percent HIV and percent tuberculosis prevalence) [28]
7	GNI per capita (US dollars) [27]
8	Rural population (percent) [28]
9	Population age structure: percent <15 y and >60 y [29]
10	Latitude (absolute value) [30]

**Figure 1:**
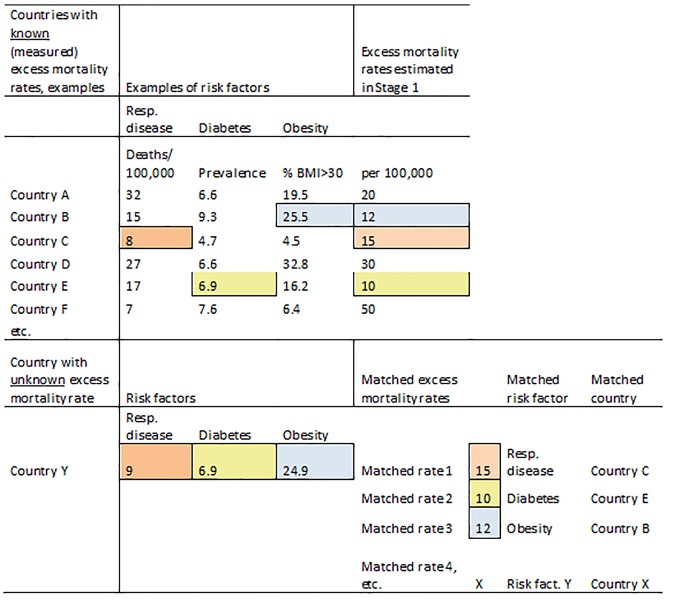
Schematic illustration of the matching method based on country indicators. BMI, body mass index; Resp. disease, respiratory disease.

Figure 1 illustrates the matching procedure for an example country (Country Y) and three example indicators.

The data analysis step of the matching method is similar to that of the multiple imputation method (below), with the same hierarchical linear random effects model but with the imputed datasets replaced by the matched datasets.

#### 4. Multiple imputation method

We chose this method to make our Stage 2 extrapolations. Like the matching method, the multiple imputation method involves two steps, a data creation step followed by a hierarchical regression modeling step to project the burden in all world countries.

In the data creation step, we used statistical correlations between the same set of country indicators that we used in the matching method (Table 3) and all the Stage 1 mortality estimates to create a distribution of possible mortality values for each unknown country in the world. We then chose a random sample of 20 possible values from each country's distribution for further analysis. Because each Stage 1 point estimate was associated with uncertainty, we repeated the data creation step for each of the Stage 1 lower and upper 95% CI bounds. Thus, the final analysis dataset contained 60 estimates per country.

In the analysis step, we applied a hierarchical linear random effects regression model [31] to the final dataset to generate a point estimate (with standard error) for each country [32],[33]. We calculated the Stage 2 mortality rates (and their confidence intervals) simultaneously (i.e., in one model) for each country, each region, and the entire globe. The procedure was undertaken for persons of all ages and separately for persons <65 y. The model used was
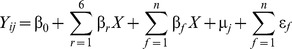
(2)where

Y = imputed individual rate

*i* = individual measurement

*j* = country (1 … 197)

μ*_j_* = between-country variance; μ*_j_*∼N(0, 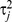)

ε_*f*_ = error variance for factor/imputed dataset, normally distributed; μ_f_∼N(0, 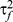)

*r* = WHO region (1 … 7); coding (*r* = 1…6), ([0,1]−1)/6; *r* = 7, countries not belonging to a region (1 if *r* = 7, else 0)

*f* = imputed datasets (1 … 20); coding ([0,1]−1)/*n*; *n* = number of factors or datasets

Estimated rates were then given by:

World = β_0_

WHO region = β_0_+β*_r_*

Country = β_0_+β*_r_*+μ*_j_*

Statistical 95% confidence intervals for these estimates were calculated using standard methods. We performed the imputation procedures with the Amelia II software package [34], and used the MLwiN version 2.1 package for the analysis model [35].

We evaluated the performance of each of the four candidate Stage 2 methods. We rejected the survey method because the results could not show regional variation, a major disadvantage given that the Stage 1 results showed considerable variation among the regions. We rejected the GNI/latitude method for two reasons. First, the estimates for our validation countries did not match the validation estimates the method produced (Table 4). Second, GNI and latitude did not result in statistically significant coefficients.

**Table 4:** External validation of GLaMOR Stage 2 projections for pandemic respiratory deaths, comparing GLaMOR Stage 2 projections for each projection method to five single-entity estimates made by collaborators using methods other than the GLaMOR regression model.

Country	Source	Estimate	95% CI	Projection Method			
				Matching	Multiple Imputation	GNI/Latitude	Survey
**All ages**							
Bangladesh	Verbal autopsy [45]	4.0	NA	2.1	4.0*	0.4	2.0
Brazil	GLaMOR; Serfling	4.3	NA	2.8	3.5*	0.1	2.0
Canada	Lab-confirmed deaths [53]	2.1	1.6–2.6	1.8*	3.1	1.0	2.0
Netherlands	Poisson regression [13]	0.9	0.3–1.5	0.9*	0.9*	0.7	2.0
Peru	GLaMOR; Serfling	6.8	NA	2.5	3.6*	0.1	2.0
**Under 65 y**							
Bangladesh	Verbal autopsy [45]	3.0	1.6–3	1.2	1.8*	2.6	1.2
Brazil	GLaMOR; Serfling	3.1	NA	2.0	2.5*	0.6	1.2
Canada	Lab-confirmed deaths [53]	1.1	0.9–1.3	1.1*	2.0	0.7	1.2
Netherlands	Poisson regression [13]	0.2	0.2–0.4	0.5*	0.9	0.6	1.2
Peru	GLaMOR; Serfling	5.2	NA	1.4	2.5*	0.5	1.2

The asterisk indicates for each country and category which of the four tested Stage 2 methods was in best agreement.

NA, not available.

### Choosing between Matching and Multiple Imputation

**Figure 2:**
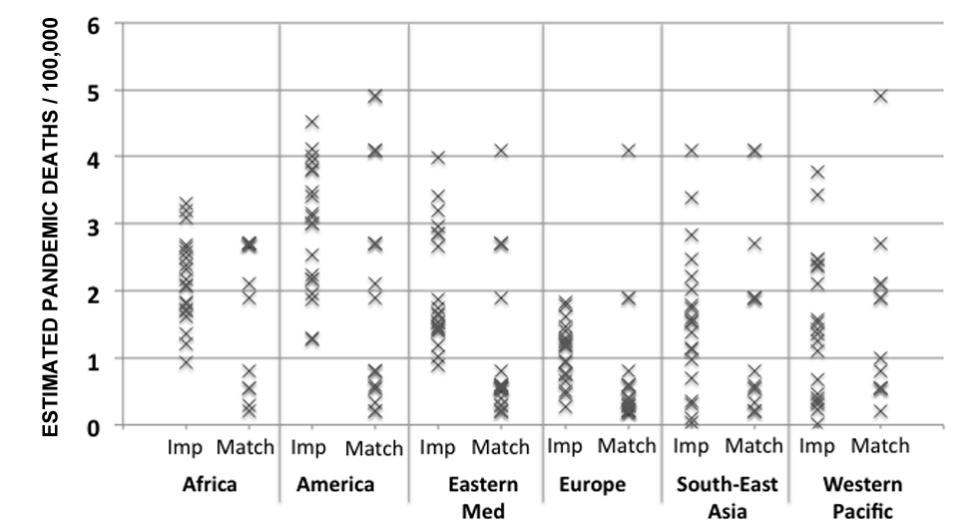
Output of the data creation phase for multiple imputation and matching for one randomly selected country per region. Eastern Med, Eastern Mediterranean; Imp, multiple imputation method; Match, matching method.

After eliminating the first two methods, only the matching and the multiple imputation methods remained. We first compared the multiple imputation and matching data creation steps for a single country in each WHO region. The results (Figure 2) demonstrate that each method produces a range of values and provides an indication of the uncertainty of the actual value. We then applied the following validation criteria to choose between the two.

#### 1. Reliability coefficients

Reliability, or internal consistency, is an important consideration. The reliability coefficient ranges from 0 to 1 (zero indicates no systematic effect) [36]. A value of 0.8 or higher indicates good (high) reliability. For the multiple imputation method the reliability coefficient for respiratory mortality was 0.78 for all ages and 0.87 for people <65 y. For the matching method the corresponding values were 0.94 and 0.96, respectively. It is important to realize, however, that this indicates only that both models had sufficient information to capture the country differences in a statistically reliable way, with no indication that one method performed better than the other.

#### 2. Stage 1 versus Stage 2 estimates

We assessed the difference between the Stage 2 single-country estimates obtained with the matching and multiple imputation methods and the original Stage 1 estimates. The differences, expressed as standard deviations, were smaller for the multiple imputation method.

#### 3. Lab-confirmed deaths as a minimum ground truth

We compared the Stage 2 estimates to country reports of the number of laboratory-confirmed H1N1pdm09 deaths (Table S1; data available for 67 countries until 31 December 2009). The GLaMOR estimates should logically be higher than the total number of laboratory-confirmed deaths in each country. Both models performed well, and the test indicated that both models had sufficient information to capture differences between countries in a statistically reliable way.

#### 4. Distribution of the predicted Stage 2 estimates

We compared the highest and lowest 20% of national estimates (both for all ages and for persons <65 y of age) derived from the multiple imputation and matching methods. For all ages, the matching method distributes the highest burden over Africa and the Americas, with 88% of the countries in the highest quintile in the Americas and 18% in Africa. The multiple imputation method distributes the highest burden not only over the Americas and Africa, but also over South-East Asia. As we have no Stage 1 estimates for South-East-Asia and only one for Africa, we cannot be sure which method is better, and this test was inconclusive.

#### 5. Comparison to five GLaMOR validation estimates

Five of the 26 Stage 1 countries (Bangladesh, Brazil, Canada, Peru, and the Netherlands) could not be analyzed with the GLaMOR Stage 1 model for various reasons. Country collaborators in Bangladesh and Canada had each generated estimates using their own methods, as national vital statistics could not be provided. For Brazil and Peru the virological data did not align with pneumonia pandemic mortality spikes (see also Schuck-Paim et al. [12]); instead we applied a classical Serfling model to regional data, which does not require virology data (similar to the approach used by Charu et al. [9]). For the Netherlands, the GLaMOR H1N1pdm09 estimates were substantially lower than those generated by the country collaborators [13]. We ran both their and our model on respiratory mortality data and found that the GLaMOR estimates were negative for the elderly, and that the upper 95% confidence interval on the all-age estimate excluded the number of laboratory-confirmed deaths. However, the Dutch model placed most H1N1pdm09 deaths in seniors (≥65 y), which was in disagreement with the age distribution in the Dutch laboratory-confirmed mortality data. Unable to reconcile these discrepancies, we accepted the country collaborators' preference for their own model results. These five country estimates (based on alternative modeling strategies) were used only to validate the Stage 2 projections. The multiple imputation method estimates were more often closer to the independent national estimates than the matching method estimates were (Table 4).

### Multiple Imputation Method Chosen

The validation tests for the most part yielded only small differences between the multiple imputation and matching methods. We selected the multiple imputation method because it produced estimates that were more consistent with those generated by country collaborators in the five GLaMOR validation countries than the estimates produced by the matching method, and because multiple imputation is an established method while the matching method was one we developed. The choice of multiple imputation had two important consequences: it resulted in systematically higher country mortality rates compared to the matching method, and it placed a higher burden in people aged 65 y and over, especially in Asia and Africa.

### Global Seasonal Burden Estimates

We computed the average global seasonal influenza burden (type A plus type B) for the years immediately prior to the pandemic. Specifically, we calculated the average pre-pandemic seasonal influenza mortality for each Stage 1 country using model parameter values from each pre-pandemic season, then projected these estimates to global and regional values using our Stage 2 multiple imputation procedure (Table S4). The number of pre-pandemic seasons available to us from each country was variable, and we made no attempt to control for differences in influenza type or subtype dominance.

### Calculating All-Age Mortality

Because of large background mortality in the elderly, it was difficult to measure all-age influenza-related mortality with precision in lower-burden countries. For example, in some European countries the H1N1pdm09 mortality impact was so subtle that the model applied to all-age time series produced a negative point estimate for the H1N1pdm09 burden, with confidence intervals that at times excluded the “ground truth” minimum of the reported number of laboratory-confirmed H1N1pdm09 deaths from that country. Modeling the data for the <65-y age group, however, almost always resulted in estimated H1N1pdm09 mortality rates that were comparable to, or far higher than, the laboratory-confirmed mortality count.

We therefore elected to generate Stage 2 all-age burden projections in two ways: one based on Stage 2 all-age estimates, and the other based on the <65-y Stage 2 estimates, which we proportionally projected to all ages using data from laboratory surveillance indicating that 85% of confirmed H1N1pdm09 deaths occurred in the younger group (Table S1).

### Sensitivity Analysis

We investigated the sensitivity of our global Stage 2 burden estimates to changes in the Stage 1 sample by successively removing one Stage 1 country from the Stage 2 input dataset and rerunning the Stage 2 model. Because the range from this analysis was always wider than the 95% confidence intervals derived from the Stage 1 and 2 statistical procedures, we chose to report this range as a more realistic view of the uncertainty (see Table 5; the statistical 95% CIs can be found in Tables S2 and S3).

**Table 5:** Global and regional GLaMOR Stage 2 projections of pandemic respiratory mortality, where all age estimates were derived both from Stage 1 all-age estimates and from the <65-y age group results adjust to 100% using the laboratory-confirmed mortality surveillance age distribution.

Region	<65 y, Stage 1		All Ages, Stage 1		All Ages (from Stage 1 <65 y)^a^	
	Estimate	Range^b^	Estimate	Range^b^	Estimate	Range^b^
World	117,130	104,450–132,080	188,660	175,280–203,250	137,800	122,882–155,388
Africa	17,922	15,408–21,172	25,476	22,431–28,447	21,085	18,127–24,908
Eastern Mediterranean	11,108	10,092–12,564	14,911	13,592–17,718	13,068	11,873–14,781
Europe	8,463	6,686–8,894	11,223	10,557–13,883	9,956	7,866–10,464
Americas	22,975	20,768–28,328	35,298	29,107–38,461	27,029	24,433–33,327
South-East Asia	30,412	25,829–36,861	73,449	50,012–83,346	35,779	30,387–43,366
Western Pacific	20,179	17,023–25,259	30,554	28,427–41,862	23,740	20,027–29,716

a Calculated assuming 85% of all deaths occurred among persons <65 y, as was the case with laboratory-confirmed pandemic deaths identified in seven countries; see Table S1.

b The confidence range was derived from a sensitivity analysis in which we successively removed one Stage 1 county at a time from the Stage 2 input set and recalculated the global and regional burden.

## Results

### Stage 1 Findings

The GLaMOR Stage 1 countries experienced one to three pandemic waves during 2009. Most H1N1pdm09 deaths occurred in winter months: November–December in the northern hemisphere and July–August in the southern hemisphere. Several Asian countries experienced an H3N2 epidemic in the months immediately before their major H1N1pdm09 wave.

**Figure 3:**
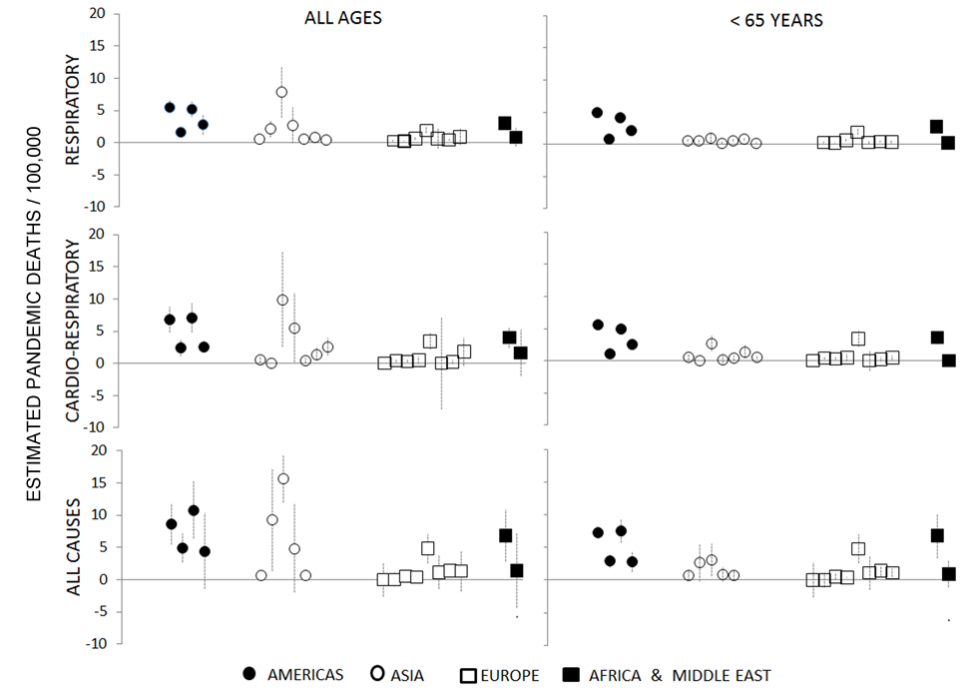
Pandemic excess mortality estimates for Stage 1 countries, by age and outcome (respiratory, cardiorespiratory, and all cause). Data are grouped into four geographical regions.

Figure 3 shows Stage 1 country H1N1pdm09 mortality rates per 100,000 population with 95% confidence intervals. The model fit (adjusted *R* ^2^) for Stage 1 respiratory mortality estimates among people <65 y was generally excellent (80%–90%), and the “lift” upon introducing the influenza virus explanatory components into the base secular model was substantial (Table 2). The pandemic burden varied considerably between countries. For countries where the burden was high, the Stage 1 model could easily separate the pandemic signal from the background noise. But where the burden was low, the H1N1pdm09 mortality point estimates became unreliable.

**Figure 4:**
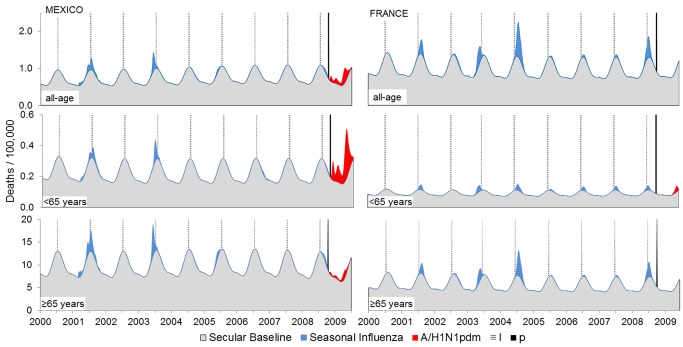
Examples of regional heterogeneity in pandemic mortality impact: Mexico (high burden) and France (low burden). In Mexico, a substantial H1N1pdm09 respiratory mortality burden (red areas above gray background mortality) occurred among children, young adults, and middle-aged persons (<65 y) of age but not among seniors (≥65 y). In France, however, there was a far less dramatic pandemic impact that, despite the similar population size, was captured only in the <65-y age group model. Seasonal influenza burden (blue areas) was also generated by the Stage 1 model. The vertical black line represents the start of the pandemic.

The various outcomes and age stratifications each provided a different balance between sensitivity and specificity. In high-burden countries such as Mexico and Argentina, the pandemic impact could be modeled with precision even for all-age time series of all-cause and cardiorespiratory mortality outcomes, as evidenced by tight 95% confidence intervals and agreement with a published Mexico study using a different Serfling regression modeling approach [9]. But in lower-burden countries, such as France and Germany, we could estimate a significant H1N1pdm09 mortality attribution only in persons <65 y and for respiratory deaths; Figure 4 illustrates the burdens in Mexico and France. Furthermore, for multiple countries we could not obtain a good model fit for finer age groups (e.g., <5, 5–14, 15–44, and 45–64 y) because of the small and fluctuating numbers of weekly deaths. We therefore chose to focus the global analysis on respiratory mortality among persons <65 y and ≥65 y, summing these point estimates to arrive at the all-age estimates.

Overall, Stage 1 respiratory mortality rates were consistently higher in the Americas, with the highest measurements in Central and South American countries. South Africa's pandemic burden was moderate and on par with that of the US and China, suggesting that Africa may have experienced a lower pandemic burden than Central and South America. In Europe, the pandemic burden was generally low and on par with national numbers of laboratory-confirmed H1N1pdm09 deaths. Spain, France, and Germany averaged a rate of just 0.3/100,000, while Romania, the lowest-income European country included in this study, felt an approximately 6-fold greater impact. In South and Central America, however, we did not find a consistent relationship between H1N1pdm09 mortality and country income group: Argentina and Mexico had particularly high pandemic death rates (∼5/100,000), whereas Chile, a country with a similar economy, had a pandemic death rate that was more than 3-fold lower.

Across all Stage 1 countries, the model placed an average of 66% of respiratory pandemic deaths in persons <65 y. However, that proportion varied widely, from 100% in several European countries, to 70%–90% in Argentina and Mexico, to less than 10% in Hong Kong and Japan. Our Stage 1 estimates for seniors (≥65 y) were considerably more uncertain than those for the <65-y age group; this was due in part to the difficulty in precisely measuring the small H1N1pdm09 burden in seniors against their high background mortality, and partly because Hong Kong and Japan were outliers with a far higher burden among seniors than all the other countries.

In the few high-burden countries where we could measure Stage 1 all-cause mortality with confidence (e.g., Argentina and Mexico), the ratio of all-cause to respiratory mortality ranged from 1.6 to 2.3.

### Stage 2 Global Projections

Using the Stage 1 model results for all ages (sum of <65-y and ≥65-y age group estimates) as input, the Stage 2 model projected a global pandemic respiratory mortality during 2009 of 189,000 (range, 175,000–203,000) deaths. These estimates correspond to an incidence rate of 2.77 (range, 2.57–2.98) per 100,000 population. Globally, 62% of these deaths were estimated to occur in persons <65 y, varying from 41% to 75% among WHO regions.

Because the pandemic virus caused considerable excess mortality in the <65-y age group, Stage 1 estimates for this age group were more reliable. To take advantage of that greater reliability, we computed the Stage 2 global and regional projections based on these estimates alone, by adjusting the <65-y estimates to all ages. To accomplish this we compiled data on the age distribution of laboratory-confirmed H1N1pdm09 deaths from mortality surveillance efforts in seven countries; these data indicated that an average of 85% of all pandemic deaths occurred in persons <65 y (Table S1). This alternative approach yielded a lower global pandemic respiratory mortality of 138,000 deaths (range, 123,000–155,000). Table 5 shows the estimated numbers of global and regional deaths obtained by both methods; mortality rates and 95% confidence intervals are given in Tables S2 and S3.

**Figure 5:**
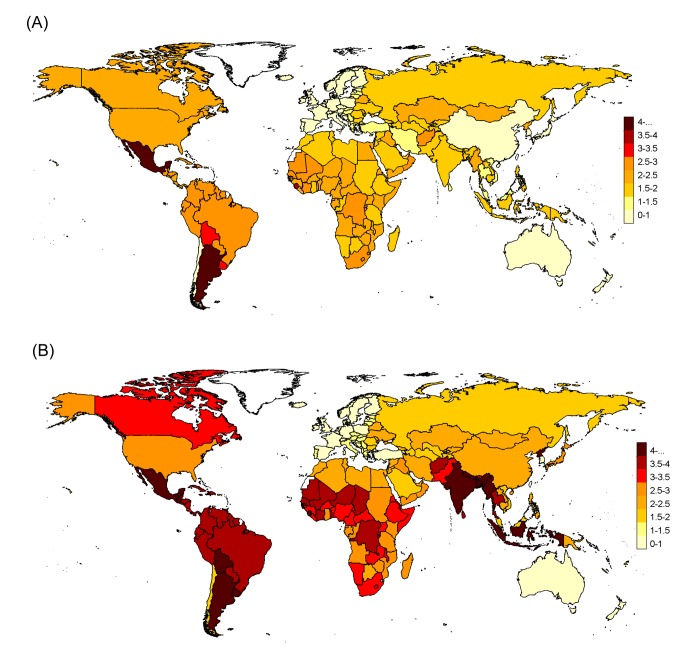
Pandemic respiratory mortality rates projected to all world countries with the Stage 2 multiple imputation model, stratified by age. (A) Under 65 y and (B) all ages. Numbers in map legend are pandemic mortality rates per 100,000 persons.

We found substantial regional heterogeneity in H1N1pdm09 mortality rates (Figure 5). The regional patterns observed among Stage 1 countries were borne out in the Stage 2 estimates, with high rates in the Americas and low rates in Europe.

**Figure 6:**
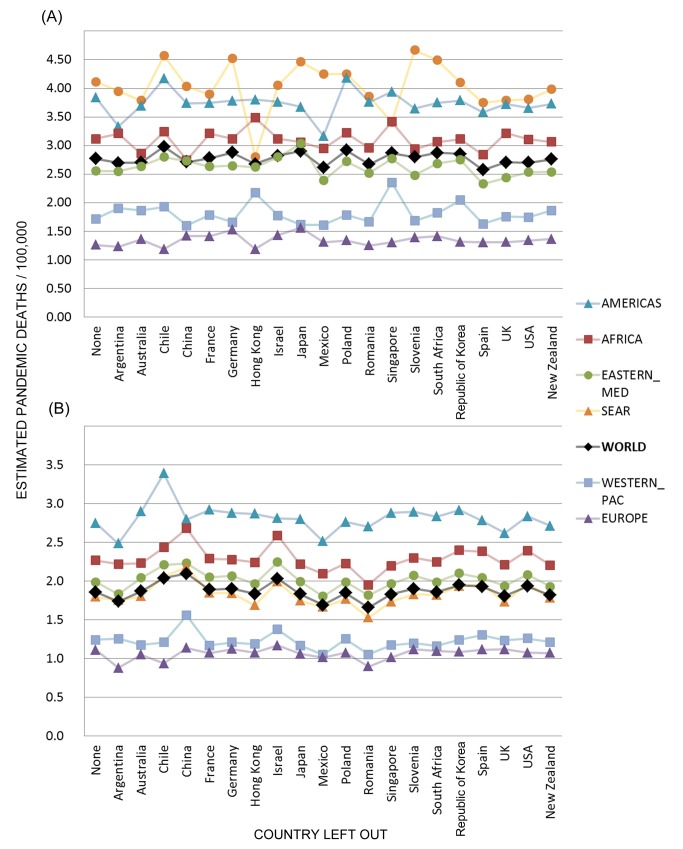
Sensitivity analysis of global and regional pandemic respiratory mortality rates. The Stage 2 model was run multiple times, each time removing one Stage 1 country, for (A) all ages and (B) <65 y. The global estimates (black diamonds) were relatively stable, but some regions were sensitive to the removal of individual countries. Figure S1 depicts the corresponding sensitivity analysis results for seasonal estimates. Eastern Med, Eastern Mediterranean; SEAR, South-East Asia; Western Pac, Western Pacific.

In the sensitivity analysis in which one Stage 1 country at a time was removed from the Stage 2 analysis, the global H1N1pdm09 mortality estimates were stable, with all-age point estimates ranging from 2.6 to 3.0 per 100,000 population from all-ages data (Figure 6A) and 1.7 to 2.1 per 100,000 from <65-y age group data (Figure 6B). However, regional estimates were more sensitive to the removal of individual countries, reflecting the importance of inclusion or exclusion of countries with “outlier” Stage 1 measurements such as Mexico and Chile in the Americas and Hong Kong in the South-East Asia region.

### Seasonal Influenza Mortality Estimates

**Figure 7:**
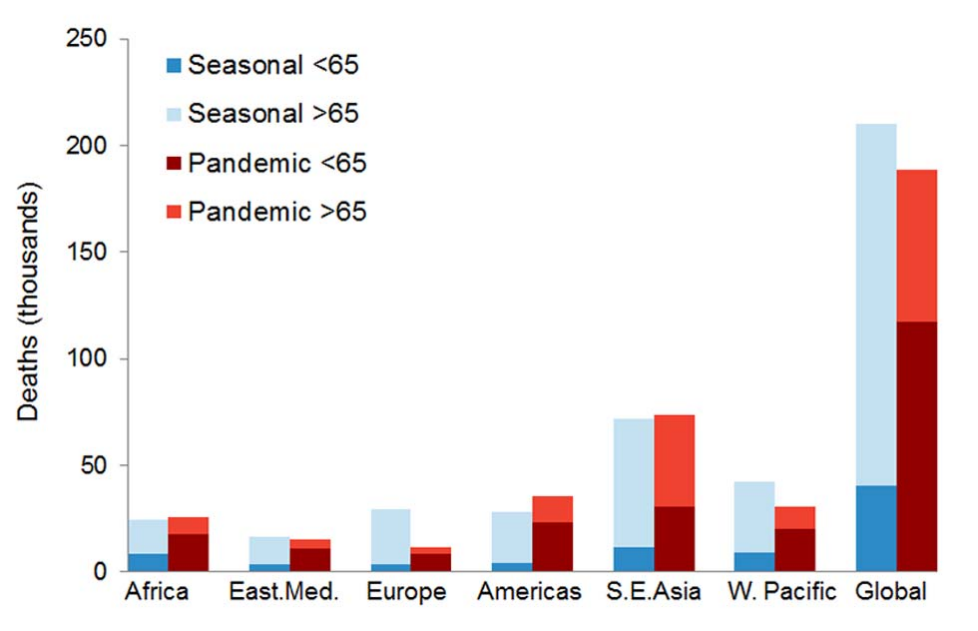
Age distribution of projected global and regional respiratory mortality, for both pandemic and seasonal influenza mortality estimates. East.Med, Eastern Mediterranean.

We generated an estimate of the average global seasonal influenza mortality burden, based on Stage 1 average seasonal influenza attributions across the pre-pandemic period. The Stage 2 multiple imputation method projected a global seasonal influenza burden of 210,000 influenza-related respiratory deaths per influenza season; of these only 19% occurred in persons <65 y of age. The range of seasonal influenza mortality estimates in the sensitivity analysis (148,000 to 249,000 deaths per year) was again wider than the 95% confidence intervals; thus we believe the range to be a better measure of uncertainty. The effect of removing individual Stage 1 countries on the seasonal influenza mortality estimates was more pronounced than for the pandemic estimates (Figure S1). Figure 7 shows the marked age shift in global and regional pandemic mortality burden toward persons aged <65 y.

## Discussion

Our analysis suggests that between 123,000 and 203,000 H1N1pdm09 respiratory deaths occurred globally in 2009. This range is derived from the all-age burden computed in two different ways: the higher figure is the upper bound of the all-age Stage 2 projection as given by the sensitivity analysis, while the lower estimate is the lower sensitivity analysis bound of the more reliable <65-y age group estimate projected to all ages.

That range places H1N1pdm09 mortality below that of previous influenza pandemics, which varied from ∼1 million deaths in 1968 to ∼50 million deaths in 1918 [37]. But the majority (62%–85%) of the H1N1pdm09 deaths occurred among persons under 65 y of age, compared to only 19% of the 148,000 to 249,000 seasonal influenza respiratory deaths per season. It is this “signature age shift” that sets pandemic influenza apart from seasonal influenza [4],[6],[38],[39]. The severity of the 2009 pandemic would therefore be better measured with more complex metrics, such as life-years lost [8], when the necessary age- and risk-stratified mortality data become available.

When the H1N1pdm09 virus first emerged, early assessments of the threat level were mixed. Events in Mexico and Argentina in April and May 2009 suggested a severe pandemic on par with the 1957 pandemic or worse [40],[41], while data from New Zealand during June through August (their winter season) revealed a mild mortality impact [42]. By applying the same Stage 1 model form to comparable mortality data across countries, we documented that large regional variability did in fact occur: the GLaMOR Stage 1 estimates revealed an almost 20-fold higher mortality impact for several countries in the Americas than for New Zealand, Australia, and most countries in Europe.

Many factors might have contributed to the regional differences in H1N1pdm09 mortality impact, including the previous influenza exposure history of the population, use of antiviral drugs, the number and duration of pandemic waves during 2009, influenza vaccination coverage in preceding seasons, access to intensive care, and use of public health mitigation strategies. Use of pandemic vaccine is not on the list as it became available too late to play a role in 2009 [43]. One intriguing possibility is that H1N1pdm09 severity might have been exacerbated by recent circulation of H1N1 viruses distantly related to the pandemic virus. This is consistent with H1N1 having predominated in the Americas and H3N2 in Europe in the 2008–2009 season. It is also supported by a recent study demonstrating enhanced H1N1pdm09 disease in piglets recently immunized with a genetically distant seasonal H1N1 vaccine [44].

Our global estimates were in reasonable agreement with those of Dawood et al. [19], to our knowledge the only other published study of global mortality from the 2009 pandemic. The GLaMOR respiratory estimate range fell within Dawood's ranges of global respiratory deaths (25th to 75th percentile, 105,700 to 395,600; 5th to 95th percentile, 39,000 to 1,315,800). However, the two studies used very different modeling strategies. Dawood et al. collected symptomatic attack rates and case fatality rates from a set of high-income countries, then applied a country-specific “respiratory mortality multiplier” proportional to the underlying risk of dying from respiratory diseases in pre-pandemic years.

**Figure 8:**
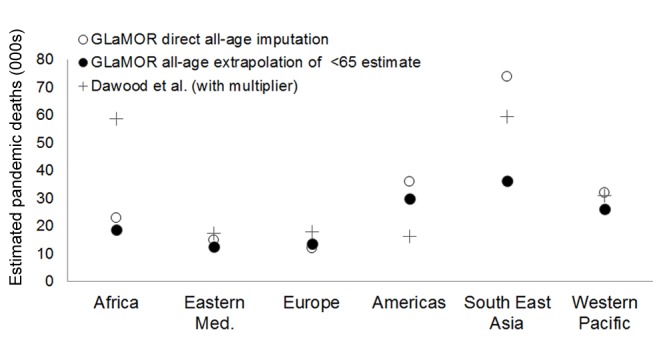
Comparison of GLaMOR mortality estimates to those of Dawood et al. GLaMOR all-age respiratory mortality estimated directly from all-age multiple imputation (open circles) and by proportional extrapolation of the <65-y age group estimate to all ages using the age distribution of laboratory-confirmed mortality surveillance (black circles), compared to estimates by Dawood et al. [19] (black plus signs). Eastern Med, Eastern Mediterranean.

But at the regional level, the two approaches produced entirely different patterns (Figure 8). For example, we measured the highest H1N1pdm09 mortality rates in the Americas, while Dawood et al. projected low mortality rates there, having set the “respiratory mortality multiplier” to 1 for that region. Furthermore, Dawood et al. projected the highest mortality in Africa, while our Stage 1 pandemic respiratory estimate for South Africa—based on actual mortality data from South Africa—was manyfold lower than Dawood et al.'s. Their projection for India and South Asia was also high, although a Bangladesh study team measured a pandemic burden that was not particularly high [45]. Finally, surveillance data indicate that H1N1pdm09 virus activity was delayed in African countries, so that most H1N1pdm09 deaths occurred in subsequent years in those countries [46].

Neither Dawood et al. nor our study had many data from Africa and South-East Asia, however, so what actually happened in these largely tropical regions remains unclear. However, a recent study demonstrated that the H1N1pdm09 mortality impact was far greater in temperate and wealthy southern regions of Brazil than in tropical and less-wealthy northern regions [12]. This unique insight from one large country that straddles climate zones suggests that the burden in the tropics was not necessarily higher than in temperate climates.

**Figure 9:**
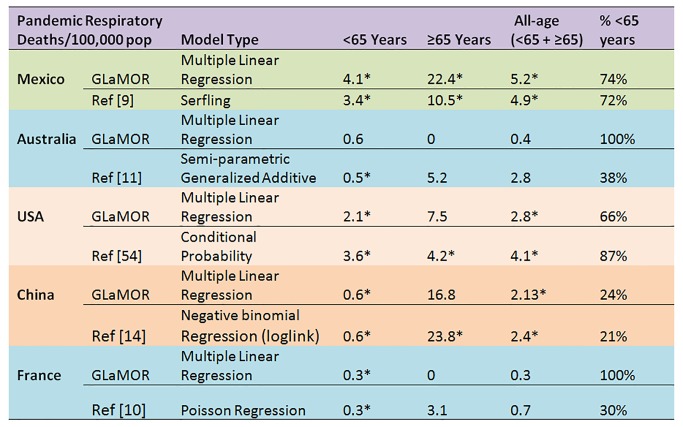
Comparison of Stage 1 modeled pandemic respiratory mortality rates, by age, to published estimates for Mexico, Australia, US, China, and France by authors using various modeling strategies. Asterisks indicate significance at the *p*<0.05 level.

**Figure 10:**
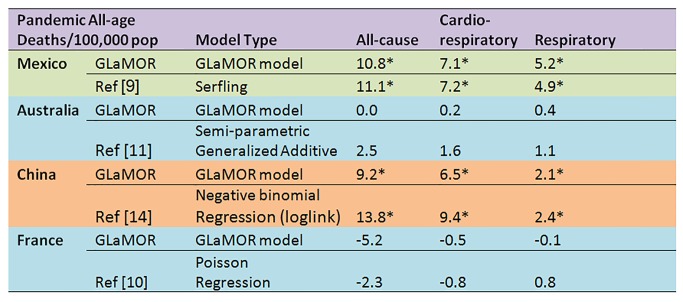
Comparison of Stage 1 modeled pandemic all-age mortality rates, by cause, to published estimates for Mexico, Australia, US, China, and France by authors using various modeling strategies. Asterisks indicate significance at the *p*<0.05 level.

The H1N1pdm09 burden estimates from a few GLaMOR participating countries have been published. As shown in Figures 9 and 10, the GLaMOR Stage 2 projections for persons <65 y are in good agreement with published estimates for Mexico, China, Australia, US, and France, despite substantial differences in modeling approaches. In contrast, the senior (≥65 y) estimates are quite variable, as were the GLaMOR all-age results.

Although our conservative range of global respiratory mortality estimates is an order of magnitude greater than the reported global number of WHO laboratory-confirmed deaths, it likely substantially understated the total H1N1pdm09 mortality burden. First, our study missed deaths that occurred late in the 2009–2010 winter as well as those occurring in later pandemic waves. For example, one-third of Germany's laboratory-confirmed first-wave deaths occurred in early 2010, while substantial waves of H1N1pmd09 mortality were observed later in the UK in 2010–2011 [47] and in Mexico in 2011–2012 [48],[49]. Second, many H1N1pdm09 deaths may not have been recorded as respiratory deaths. Furthermore, we were forced to model “underlying cause of death” data, which may be biased towards underlying chronic disease and thus undercount respiratory deaths. Moreover, we refrained from generating a global estimate based on all-cause or cardiorespiratory deaths (the latter a preferred outcome for measuring seasonal influenza burden), since we were unable to detect significant pandemic excess mortality for these outcomes in most Stage 1 countries. We agree with Lemaitre et al. [10] that all-cause mortality is not a useful outcome for assessing H1N1pdm09 mortality in mild impact countries. However, in the few high-burden countries where we could measure all-cause mortality with confidence (e.g., Argentina and Mexico), the ratio of all-cause to respiratory mortality was ∼2∶1; Charu et al. reported a similar ratio for Mexico [9]. If this 2∶1 ratio pertained globally, the global pandemic all-cause mortality burden would have been about 300,000–400,000 deaths, approximately double our range of respiratory estimates.

Our approach had several strengths. Because the analysis was based on the pandemic “excess” mortality that actually occurred in 20 Stage 1 countries in 2009, we were able to map large and important regional differences in the H1N1pdm09 mortality burden that had not been captured in a previous study [19]. Because our collaborators contributed several years of data, we also were able to generate a global estimate of average seasonal influenza mortality, to which we could compare the pandemic burden. And because our single-country Stage 1 estimates were based on widely used analytical methods, our H1N1pdm09 mortality burden estimates are comparable to estimates made for historic influenza pandemics.

We recognize several caveats of our study, however. First, we lacked good representation of low-income countries and countries in South-East Asia, the Eastern Mediterranean, and Africa. Second, we were unable to explain the substantial pandemic mortality attributions among seniors that we and others measured in Hong Kong [50] and Japan, a pattern very unlike that documented by laboratory-confirmed mortality surveillance efforts in multiple countries globally (Table S1). Our Hong Kong collaborators maintained that seniors could have been missed in the laboratory-confirmed surveillance effort, while our Japanese collaborators argued that their pandemic mortality surveillance system was not age biased. Thus, the high measured burden among seniors may be real, or a mis-measurement due to an inability of statistical models to fully control for H3N2 co-circulation in 2009 in some Asian settings. In any event, we could not resolve the issue. We therefore also generated global and regional estimates based on the less contentious <65-y age group results. Third, we might not have accounted sufficiently for spatial dependency between countries and the likely spillover effect of influenza. And finally, the seasonal estimates were not ideal because Stage 1 countries contributed data from a variable number of seasons, and not all modeling issues were fully resolved (e.g., we noted some degree of misalignment between influenza virology and seasonal pneumonia mortality peaks in some countries).

Health care policy decisions depend on reliable and timely data whereby the risks and cost-effectiveness of interventions can be evaluated. In the GLaMOR study we developed methods whereby we can make robust and comparable mortality estimates in any future pandemic. But the lack of timeliness of such reports must be remedied. Ideally, a set of sentinel countries with timely hospitalization and/or mortality data could form a global sentinel system measuring severity, provided a common protocol was in place to allow comparisons across settings. EuroMOMO (European Monitoring of Excess Mortality for Public Health Action), which collects timely age-specific all-cause mortality data in European countries, is a big step in the right direction [51]. Also Mexico, Hong Kong, and New Zealand should be lauded for timely surveillance systems that captured hospitalization, case fatality, and mortality impact in a large segment of their populations in near real time [42],[49],[52] and Canada for its timely investigations into unexpected effects of seasonal vaccines on the pandemic [55]. Going forward, these and other countries and existing networks should partner to collaboratively and rapidly assess the severity of future pandemic threats.

## Supporting Information

Figure S1 **Sensitivity analysis of the multiple imputation method seasonal estimates.** We carried out the same sensitivity analysis on the seasonal estimates—leaving one country out at a time—that we had done on the pandemic estimate. The seasonal estimates were much more sensitive to the exclusion of a country than the pandemic estimates (Figure 6). The exclusion of China had a particularly large impact on the all-age seasonal excess respiratory mortality estimate.
(TIF)

Figure S2 **A map showing WHO regions.**
(TIF)

Table S1 **Age distribution of laboratory-confirmed H1N1pdm09 deaths from surveillance efforts in seven countries.**
(DOCX)

Table S2 **Global and regional GLaMOR Stage 2 projections of pandemic respiratory mortality (numbers of deaths) with 95% CIs and sensitivity analysis ranges.**
(DOCX)

Table S3 **Global and regional GLaMOR Stage 2 projections of pandemic respiratory mortality rates with 95% CIs and sensitivity analysis ranges.**
(DOCX)

Table S4 **Results from the hierarchical linear random effects regression model for the all-age and <65-y models.**
(DOCX)

## Abbreviations

GLaMOR: Global Pandemic Mortality
GNI: gross national income
H1N1pdm09: 2009 influenza A H1N1 pandemic
ICD-10: International Classification of Diseases–10
WHO: World Health Organization
