# Supplementary figures and images for: Global Mortality Estimates for the 2009 Influenza Pandemic from the GLaMOR Project: A Modeling Study
Source: PLoS Med. 2013 Nov 26;10(11):e1001558. doi: 10.1371/journal.pmed.1001558 (PMC3841239; doi:10.1371/journal.pmed.1001558)

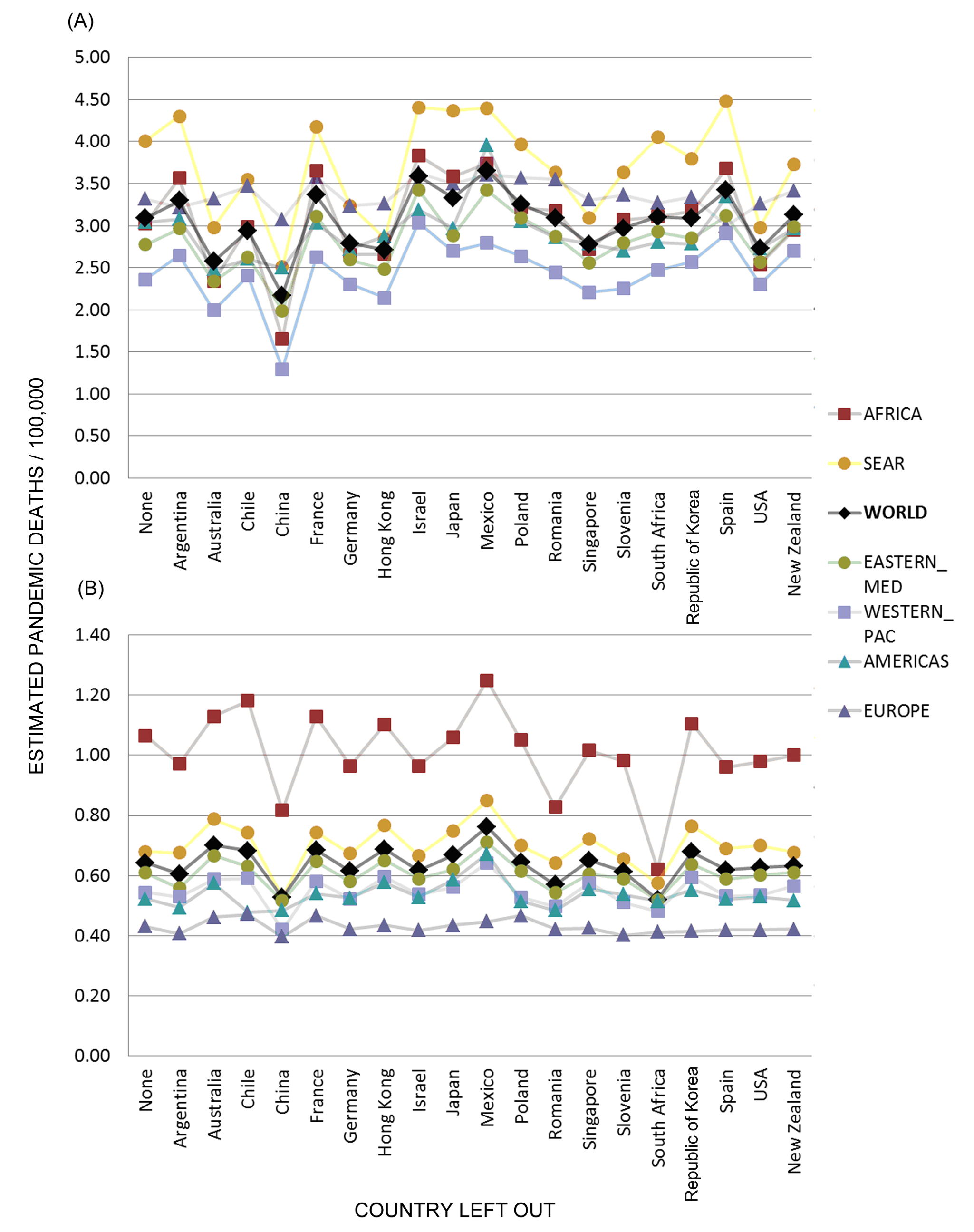

Supplement: Figure S1 — Sensitivity analysis of the multiple imputation method seasonal estimates. We carried out the same sensitivity analysis on the seasonal estimates—leaving one country out at a time—that we had done on the pandemic estimate. The seasonal estimates were much more sensitive to the exclusion of a country than the pandemic estimates (Figure 6). The exclusion of China had a particularly large impact on the all-age seasonal excess respiratory mortality estimate. (TIF) [file pmed.1001558.s001.tif]

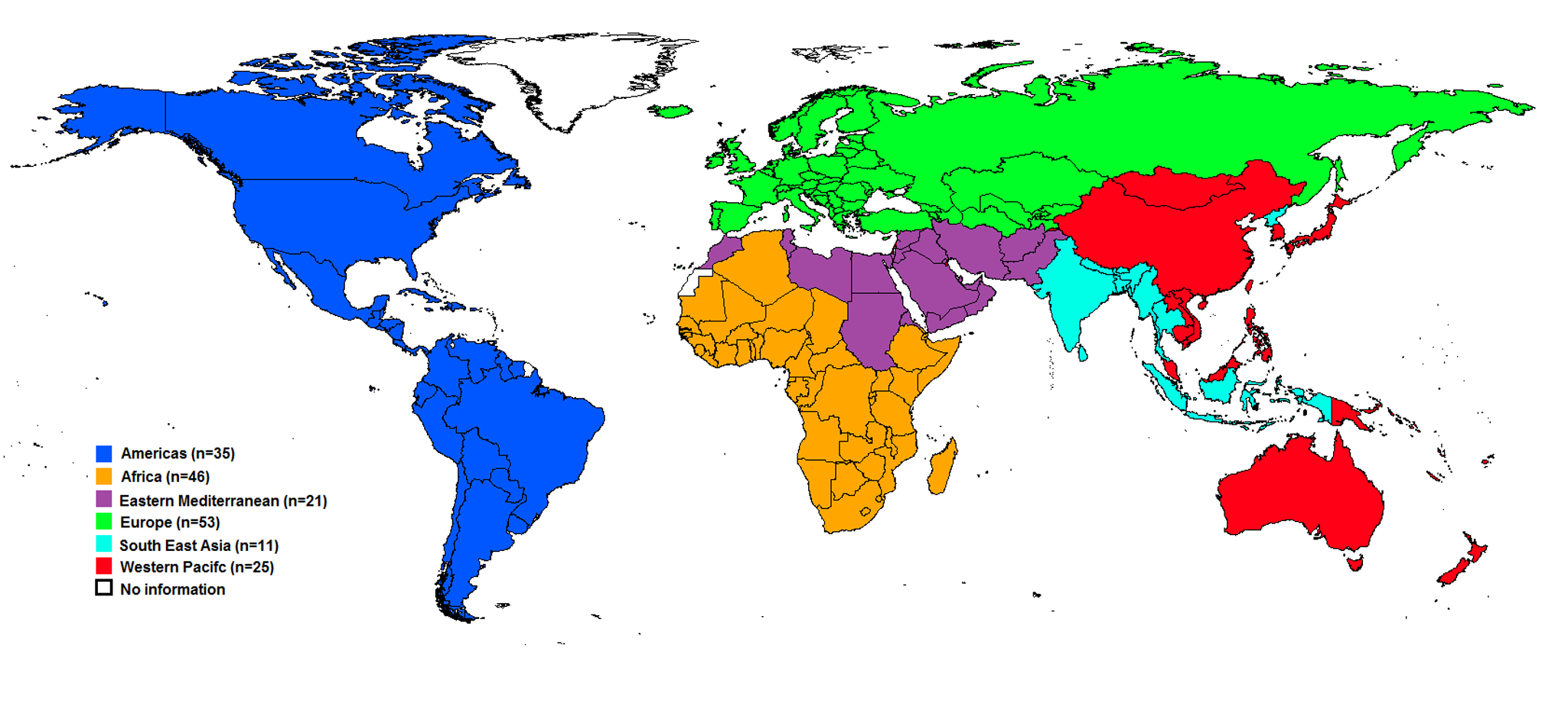

Supplement: Figure S2 — A map showing WHO regions. (TIF) [file pmed.1001558.s002.tif]
