# Supplementary material for: Global Mortality Estimates for the 2009 Influenza Pandemic from the GLaMOR Project: A Modeling Study
Source: PLoS Med. 2013 Nov 26;10(11):e1001558. doi: 10.1371/journal.pmed.1001558 (PMC3841239; doi:10.1371/journal.pmed.1001558)
Supplement: Table S1 — Age distribution of laboratory-confirmed H1N1pdm09 deaths from surveillance efforts in seven countries. (DOCX) [file pmed.1001558.s003.docx]

**Table S1.** Age distribution of laboratory-confirmed H1N1pdm09 deaths from surveillance efforts in 7 countries

|  |  | **Age** | **0-64** | | **65+** | | **Un-known** | **Total** | |
| --- | --- | --- | --- | --- | --- | --- | --- | --- | --- |
| **Region** | **Country** |  | **N** | **%** | **N** | **%** | **N** | **N** | **%** |
| Africa | South Africa |  | 90 | 97 | 3 | 3 |  | 93 | 100 |
| Americas | Canada |  | 312 | 74 | 111 | 26 | 5 | 428 | 100 |
| Americas | Brazil* |  | 1944 | 89 | 235 | 11 |  | 2179 |  |
| Europe | Germany |  | 217 | 84 | 41 | 16 |  | 258 | 100 |
| South East Asia | India (Pune)* |  | 76 | 99 | 1 | 1 |  | 77 | 100 |
| Western Pacific | China* |  | 615 | 77 | 184 | 23 |  | 799 | 100 |
| Western Pacific | Japan* |  | 153 | 77 | 45 | 23 |  | 198 | 100 |
| **Total** |  |  | **3407** | **85** | **620** | **15** |  | **4032** | **100** |

* Japan age groups 0-69 and 70+ years; China age groups 0-50 and 51+ years; India, Brazil: age groups 0-60 and 60+ years

Sources: Brazil: Authors’ calculation based on Schuck-Paim et al [[1](#_ENREF_1)] and Brazilian census data; India: Mishra et al[[2](#_ENREF_2)]; China: Liang et al[[3](#_ENREF_3)]; South Africa: Schoub [[4](#_ENREF_4)]; Data for Japan, South Africa and Germany were contributed by the country collaborator teams.

1. Schuck-Paim C, Viboud C, Simonsen L, Miller MA, Moura FE, et al. Were equatorial regions less affected by the 2009 influenza pandemic? The Brazilian experience. PLoS One 7: e41918.

2. Mishra AC, Chadha MS, Choudhary ML, Potdar VA (2010) Pandemic influenza (H1N1) 2009 is associated with severe disease in India. PLoS One 5: e10540.

3. Liang W, Feng L, Xu C, Xiang N, Zhang Y, et al. Response to the first wave of pandemic (H1N1) 2009: experiences and lessons learnt from China. Public Health 126: 427-436.

4. Schoub BD (2009) Pandemic influenza (H1N1) 2009 (swine flu). S Afr Med J 99: 576-577.
