# Supplementary material for: Global Mortality Estimates for the 2009 Influenza Pandemic from the GLaMOR Project: A Modeling Study
Source: PLoS Med. 2013 Nov 26;10(11):e1001558. doi: 10.1371/journal.pmed.1001558 (PMC3841239; doi:10.1371/journal.pmed.1001558)
Supplement: Table S2 — Global and regional GLaMOR Stage 2 projections of pandemic respiratory mortality (numbers of deaths) with 95% CIs and sensitivity analysis ranges. (DOCX) [file pmed.1001558.s004.docx]

**Table S2.** Global and regional GLaMOR Stage 2 projections of pandemic respiratory mortality numbers of deaths with 95% CIs and sensitivity analysis ranges

| **Age group** | **Region** | **Estimate** | **95% CI** | **Sensitivity analysis range** |
| --- | --- | --- | --- | --- |
| **0-65 years** |  |  |  |  |
|  | WORLD | 117,130 | (111880 - 122390) | (104450 - 132080) |
|  | WHO_AFRICA | 17,922 | (16770 - 19073) | (15408 - 21172) |
|  | WHO_EASTERN_MED | 11,108 | (9915 - 12301) | (10092 - 12564) |
|  | WHO_EUROPE | 8,463 | (7410 - 9516) | (6686 - 8894) |
|  | WHO_AMERICAS | 22,975 | (21531 - 24419) | (20768 - 28328) |
|  | WHO_SEAR | 30,412 | (25426 - 35399) | (25829 - 36861) |
|  | WHO_WESTERN_PAC | 20,179 | (16628 - 23729) | (17023 - 25259) |
| **All-ages, as the sum of <65 and ≥65** | |  |  |  |
|  | WORLD | 188,660 | (181140 - 196170) | (175280 - 203250) |
|  | WHO_AFRICA | 25,476 | (23902 - 27049) | (22431 - 28447) |
|  | WHO_EASTERN_MED | 14,911 | (13265 - 16558) | (13592 - 17718) |
|  | WHO_EUROPE | 11,223 | (9597 - 12849) | (10557 - 13883) |
|  | WHO_AMERICAS | 35,298 | (33198 - 37397) | (29107 - 38461) |
|  | WHO_SEAR | 73,449 | (66496 - 80402) | (50012 - 83346) |
|  | WHO_WESTERN_PAC | 30,554 | (25409 - 35698) | (28427 - 41862) |
| **All-age from <65 age group Stage 2 estimate, proportionally adjusting for 85% of lab confirmed deaths occurring in <65** | | | | |
|  | WORLD | 137,800 | (131624-143988) | (122882 - 155388) |
|  | WHO_AFRICA | 21,085 | (19729-22439) | (18127 - 24908) |
|  | WHO_EASTERN_MED | 13,068 | (11665-14472) | (11873 - 14781) |
|  | WHO_EUROPE | 9,956 | (8718-11195) | (7866 - 10464) |
|  | WHO_AMERICAS | 27,029 | (25331-28728) | (24433 - 33327) |
|  | WHO_SEAR | 35,779 | (29913-41646) | (30387 - 43366) |
|  | WHO_WESTERN_PAC | 23,740 | (19562-27916) | (20027 - 29716) |

.
