# Supplementary material for: Global Mortality Estimates for the 2009 Influenza Pandemic from the GLaMOR Project: A Modeling Study
Source: PLoS Med. 2013 Nov 26;10(11):e1001558. doi: 10.1371/journal.pmed.1001558 (PMC3841239; doi:10.1371/journal.pmed.1001558)
Supplement: Table S3 — Global and regional GLaMOR Stage 2 projections of pandemic respiratory mortality rates with 95% CIs and sensitivity analysis ranges. (DOCX) [file pmed.1001558.s005.docx]

**Table S3.** Global and regional GLaMOR Stage 2 projections of pandemic respiratory mortality rates with 95% CIs and sensitivity analysis ranges.

| **Age group** | **Region** | **Estimate** | **95% CI** | **Sensitivity analysis range** |
| --- | --- | --- | --- | --- |
| **0-65 years** |  |  |  |  |
|  | WORLD | 1.86 | (1.78-1.94) | (1.66-2.10) |
|  | WHO_AFRICA | 2.27 | (2.12-2.41) | (1.95-2.68) |
|  | WHO_EASTERN_MED | 1.98 | (1.77-2.20) | (1.80-2.25) |
|  | WHO_EUROPE | 1.11 | (0.97-1.25) | (0.88-1.17) |
|  | WHO_AMERICAS | 2.75 | (2.57-2.92) | (2.48-3.39) |
|  | WHO_SEAR | 1.80 | (1.50-2.09) | (1.53-2.18) |
|  | WHO_WESTERN_PAC | 1.24 | (1.02-1.46) | (1.05-1.55) |
| **All-ages, as the sum of <65 and ≥65** | |  |  |  |
|  | WORLD | 2.77 | (2.66-2.88) | (2.57-2.98) |
|  | WHO_AFRICA | 3.12 | (2.93-3.31) | (2.75-3.48) |
|  | WHO_EASTERN_MED | 2.55 | (2.27-2.84) | (2.33-3.03) |
|  | WHO_EUROPE | 1.26 | (1.08-1.44) | (1.19-1.56) |
|  | WHO_AMERICAS | 3.84 | (3.61-4.07) | (3.17-4.18) |
|  | WHO_SEAR | 4.11 | (3.73-4.50) | (2.80-4.67) |
|  | WHO_WESTERN_PAC | 1.72 | (1.43-2.01) | (1.60-2.35) |
| **All-age from <65 age group Stage 2 estimate, proportionally adjusting for 85% of lab confirmed deaths occurring in <65** | | | | |
|  | WORLD | 2.19 | (2.09 - 2.29) | (1.95 - 2.47) |
|  | WHO_AFRICA | 2.67 | (2.49 - 2.83) | (2.29 - 3.15) |
|  | WHO_EASTERN_MED | 2.34 | (2.08 - 2.59) | (2.12 - 2.64) |
|  | WHO_EUROPE | 1.31 | (1.14 - 1.47) | (1.03 - 1.37) |
|  | WHO_AMERICAS | 3.23 | (3.02 - 3.44) | (2.92 - 3.99) |
|  | WHO_SEAR | 2.11 | (1.77 - 2.46) | (1.80 - 2.56) |
|  | WHO_WESTERN_PAC | 1.46 | (1.20 - 1.72) | (1.24 - 1.83) |
