# Supplementary material for: Global Mortality Estimates for the 2009 Influenza Pandemic from the GLaMOR Project: A Modeling Study
Source: PLoS Med. 2013 Nov 26;10(11):e1001558. doi: 10.1371/journal.pmed.1001558 (PMC3841239; doi:10.1371/journal.pmed.1001558)
Supplement: Table S4 — Results from the hierarchical linear random effects regression model for the all-age and <65-y models. (DOCX) [file pmed.1001558.s006.docx]

**Table S4**. Results from the hierarchical linear random effects regression model for all age and under 65 years models*.

|  | **ALL AGE** | | **UNDER 65** | |
| --- | --- | --- | --- | --- |
| **Fixed effects** | **Estimate (standard error)** | **P value** | **Estimate (standard error)** | **P value** |
| Intercept world | 2.767(0.056) | 0.000 | 1.858(0.043) | 0.000 |
| Region (rest) | 1.429(0.247) | 0.000 | 0.401(0.187) | 0.032 |
| Region Africa | 1.403(0.177) | 0.000 | 1.026(0.134) | 0.000 |
| Region east-med | 0.837(0.206) | 0.000 | 0.743(0.156) | 0.000 |
| Region Europe | -0.455(0.174) | 0.009 | -0.131(0.132) | 0.321 |
| Region America | 2.124(0.188) | 0.000 | 1.506(0.142) | 0.000 |
| Region SEAR | 2.399(0.247) | 0.000 | 0.555(0.187) | 0.003 |
| Factor 2 | 0.091(0.095) | 0.338 | -0.012(0.053) | 0.816 |
| Factor 3 | 0.066(0.091) | 0.471 | -0.006(0.052) | 0.915 |
| Factor 4 | 0.063(0.095) | 0.506 | 0.025(0.053) | 0.629 |
| Factor 5 | 0.032(0.093) | 0.728 | 0.007(0.053) | 0.900 |
| Factor 6 | 0.129(0.097) | 0.184 | -0.046(0.053) | 0.380 |
| Factor 7 | -0.058(0.091) | 0.521 | 0.042(0.053) | 0.432 |
| Factor 8 | 0.089(0.094) | 0.341 | -0.013(0.051) | 0.794 |
| Factor 9 | 0.043(0.095) | 0.649 | 0.028(0.053) | 0.595 |
| Factor 10 | 0.171(0.093) | 0.066 | -0.062(0.053) | 0.246 |
| Factor 11 | 0.078(0.093) | 0.397 | 0.007(0.054) | 0.898 |
| Factor 12 | 0.085(0.094) | 0.365 | 0.026(0.053) | 0.629 |
| Factor 13 | 0.046(0.095) | 0.629 | 0.006(0.054) | 0.911 |
| Factor 14 | -0.041(0.092) | 0.658 | -0.045(0.053) | 0.403 |
| Factor 15 | 0.014(0.093) | 0.877 | -0.002(0.051) | 0.968 |
| Factor 16 | 0.058(0.096) | 0.549 | -0.036(0.054) | 0.496 |
| Factor 17 | 0.119(0.093) | 0.199 | 0.021(0.052) | 0.688 |
| Factor 18 | -0.067(0.090) | 0.458 | 0.036(0.053) | 0.498 |
| Factor 19 | 0.092(0.095) | 0.334 | -0.005(0.053) | 0.921 |
| Factor 20 | 0.030(0.093) | 0.749 | -0.027(0.052) | 0.607 |
|  | | | | |
| **Random effects** | **Variance(standard error)** | **P value** | **Variance(standard error)** | **P value** |
| Between countries | 0.394(0.045) | 0.000 | 0.235(0.025) | 0.000 |
| Factor 1 error | 2.552(0.153) | 0.000 | 0.783(0.047) | 0.000 |
| Factor 2 error | 2.602(0.156) | 0.000 | 0.830(0.050) | 0.000 |
| Factor 3 error | 2.201(0.133) | 0.000 | 0.772(0.046) | 0.000 |
| Factor 4 error | 2.564(0.154) | 0.000 | 0.790(0.048) | 0.000 |
| Factor 5 error | 2.371(0.143) | 0.000 | 0.810(0.049) | 0.000 |
| Factor 6 error | 2.824(0.169) | 0.000 | 0.805(0.048) | 0.000 |
| Factor 7 error | 2.134(0.129) | 0.000 | 0.811(0.049) | 0.000 |
| Factor 8 error | 2.472(0.149) | 0.000 | 0.697(0.042) | 0.000 |
| Factor 9 error | 2.634(0.158) | 0.000 | 0.808(0.049) | 0.000 |
| Factor 10 error | 2.374(0.143) | 0.000 | 0.828(0.050) | 0.000 |
| Factor 11 error | 2.332(0.140) | 0.000 | 0.864(0.052) | 0.000 |
| Factor 12 error | 2.472(0.149) | 0.000 | 0.818(0.049) | 0.000 |
| Factor 13 error | 2.556(0.154) | 0.000 | 0.894(0.054) | 0.000 |
| Factor 14 error | 2.247(0.135) | 0.000 | 0.841(0.051) | 0.000 |
| Factor 15 error | 2.400(0.144) | 0.000 | 0.711(0.043) | 0.000 |
| Factor 16 error | 2.741(0.165) | 0.000 | 0.851(0.051) | 0.000 |
| Factor 17 error | 2.344(0.141) | 0.000 | 0.778(0.047) | 0.000 |
| Factor 18 error | 2.080(0.125) | 0.000 | 0.824(0.050) | 0.000 |
| Factor 19 error | 2.638(0.158) | 0.000 | 0.807(0.049) | 0.000 |
| Factor 20 error | 2.363(0.142) | 0.000 | 0.758(0.046) | 0.000 |

*Each factor represents an imputed country dataset, see the formula of the data analysis step in the main manuscript, where f stands for imputed dataset. “Factor n” in the table above represents the term and “Factor n error” represents $\varepsilon_{f}$.


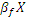

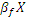


The Stage 2 model contains many components, many of which are nuisance parameters used to control for bias and to adjust for the way the datasets are created. Useful fixed effects components are the ‘Intercept world,’ which yields the global rate, and the ‘Regional’ parameters (e.g. Africa and Eastern Mediterranean), which yield the regional rates, and the ‘Between countries’ variance, which yields the random effects component. Note that overall fit statistics (e.g. R^2^) have little meaning here due to the large number of nuisance parameters; we used the reliability coefficient instead.
